# Closure of spontaneous cerebrospinal fluid leakage via fistula at the lateral wall of the sphenoid sinus using a bone pile

**DOI:** 10.1002/ccr3.5510

**Published:** 2022-03-03

**Authors:** Kosuke Takabayashi, Shuho Gotoh, Maeho Yamasaki, Katsumi Takizawa

**Affiliations:** ^1^ Department of Otorhinolaryngology Japanese Red Cross Asahikawa Hospital Hokkaido Japan; ^2^ Department of Neurosurgery Japanese Red Cross Asahikawa Hospital Hokkaido Japan

**Keywords:** high‐resolution computed tomography, pile‐driving, rigid, skull base, transseptal approach

## Abstract

This report describes a case of spontaneous cerebrospinal fluid leak through a narrow canal‐like fistula in the lateral wall of the sphenoid sinus, which was managed through rigid reconstruction. Rigid reconstruction of the skull base was performed by fitting a pile‐shaped bone into the fistula like the pile‐driving technique.

## Graphical abstract

The cranial base defect that causes spontaneous cerebrospinal fluid leakage is sometimes narrow and canal‐like, requiring a detailed investigation of the sphenoid sinus. Rigid reconstruction is possible even in such cases.

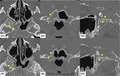

## 1 INTRODUCTION

Spontaneous cerebrospinal fluid (CSF) leakage occurs because of increased intracranial pressure; however, it has been reported to be caused by residual Sternberg's canal^1^ and progressive pneumatization of the lateral fossa of the sphenoid sinus. The increased intracranial pressure leads to the formation of arachnoid pits, which leads to progressive bone destruction of the skull base.^2^, ^3^, ^4^, ^5^, ^6^ Therefore, skull base reconstruction should be multilayered and as strong as possible.

Despite various techniques reported for multilayer reconstruction of the skull base, small defects are typically reconstructed using fat or fascia, while rigid reconstruction has not been reported.^7^, ^8^, ^9^

In this report, we describe a novel technique for managing fistulas of the lateral wall of the sphenoid sinus through rigid reconstruction in which a bone pile was formed and inserted similar to the pile‐driving technique. This technique is effective in reconstructing thin and long fistula‐like bone defects, where rigid reconstruction has not been considered.

## 2 CASE REPORT

**FIGURE 1:**
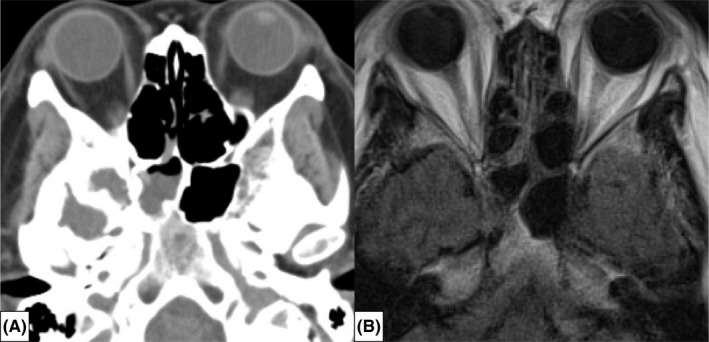
Brain images at the first examination. (A) Brain computed tomography (CT) with 4‐mm slice thickness showing fluid accumulation with air inclusion in the right sphenoid sinus. No fistula is detected. (B) Fluid‐attenuated inversion recovery magnetic resonance image (MRI) showing no evidence of deviation of the cranial contents into the sphenoid sinus

**FIGURE 2:**
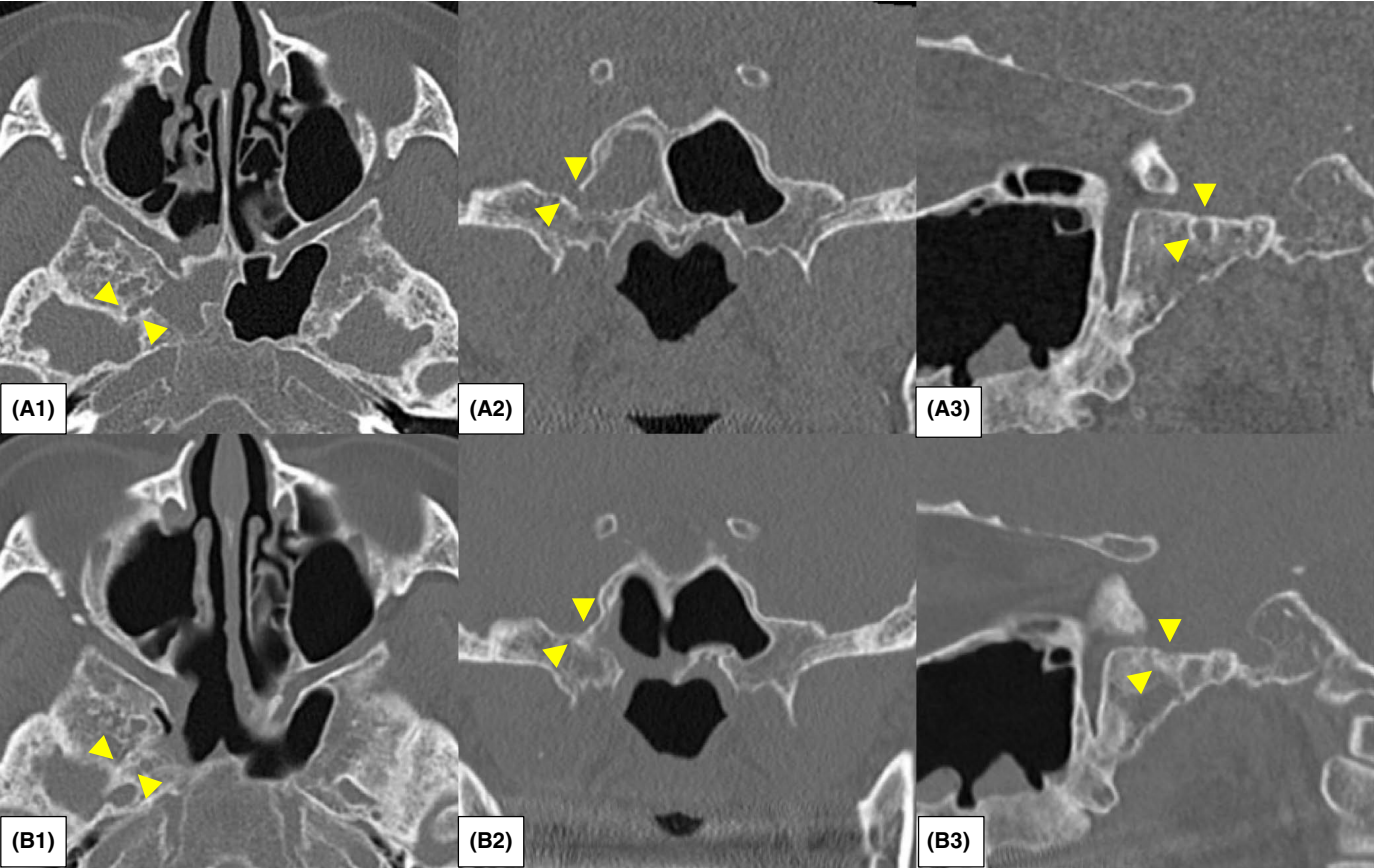
Comparison of pre‐ and postoperative CT images. (A) Preoperative high‐resolution (HR) CT images: A1, axial section; A2, coronal section; A3, sagittal section. The yellow arrowheads point to the fistula. (B) Postoperative HRCT images: A1, axial section; A2, coronal section; A3, sagittal section. The yellow arrowheads point to the fistula. The fistula is filled with bone pile

**FIGURE 3:**
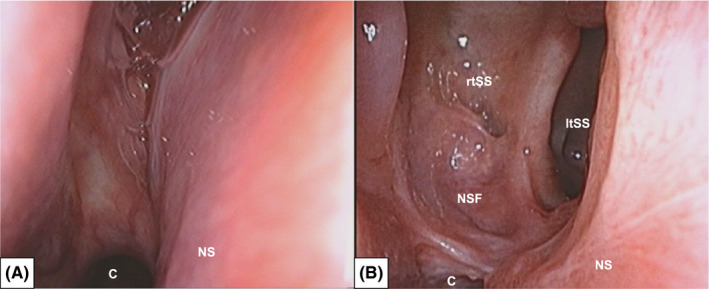
Comparison of pre‐ and postoperative endoscopic findings. (A) Preoperative endoscopic finding. Transparent serous fluid is observed to be flowing from the natural ostium of the right sphenoid sinus in the direction of the right choana. (B) Postoperative endoscopic finding. The anterior wall of the sphenoid sinus is removed. The bottom and lateral wall of the sphenoid sinus is covered by the nasoseptal mucosal flap. Abbreviations: C, choana; ltSS, left sphenoid sinus; NS, nasal septum; NSF, nasoseptal mucosal flap; rtSS, right sphenoid sinus

A 53‐year‐old woman, who had been suffering from serous rhinorrhea for several years, with no specific medical conditions except for obesity with a body mass index of 41 kg/m^2^, presented emergently to the neurological department with high fever and impaired consciousness. Cerebrospinal fluid (CSF) examination by lumbar puncture revealed a diagnosis of bacterial meningitis. Only an accumulation of fluid with air was noted in the right sphenoid sinus through brain computed tomography (CT) taken at 4‐mm slice thickness (Figure 1A) and magnetic resonance imaging (MRI) (Figure 1B). There were no other findings that could be considered a focus of inflammation. She was referred to the otorhinolaryngological department to investigate whether the right sphenoid sinus was the focus of the meningitis. High‐resolution (HR) CT images were taken with 0.5‐mm slice thickness, and coronal and sagittal sections were reconstructed (Figure 2A1‐3). HRCT revealed a skull base fistula at the lateral wall of the right sphenoid sinus and arachnoid pits at the middle cranial fossa. In addition, the fluid in the right sphenoid sinus, draining into the nasal cavity via the natural ostium of the right sphenoid sinus (Figure 3A), was collected and measured for glucose, and was found to be similar to the CSF from the lumbar region. Therefore, we diagnosed the patient with spontaneous CSF leakage due to fistula of the middle cranial fossa and an ascending bacterial infection spilled over into the cranium via the fistula. After improvement of the meningitis by medical treatment, an interdisciplinary team of otorhinolaryngologists and neurosurgeons performed reconstruction of the skull base.

**FIGURE 4:**
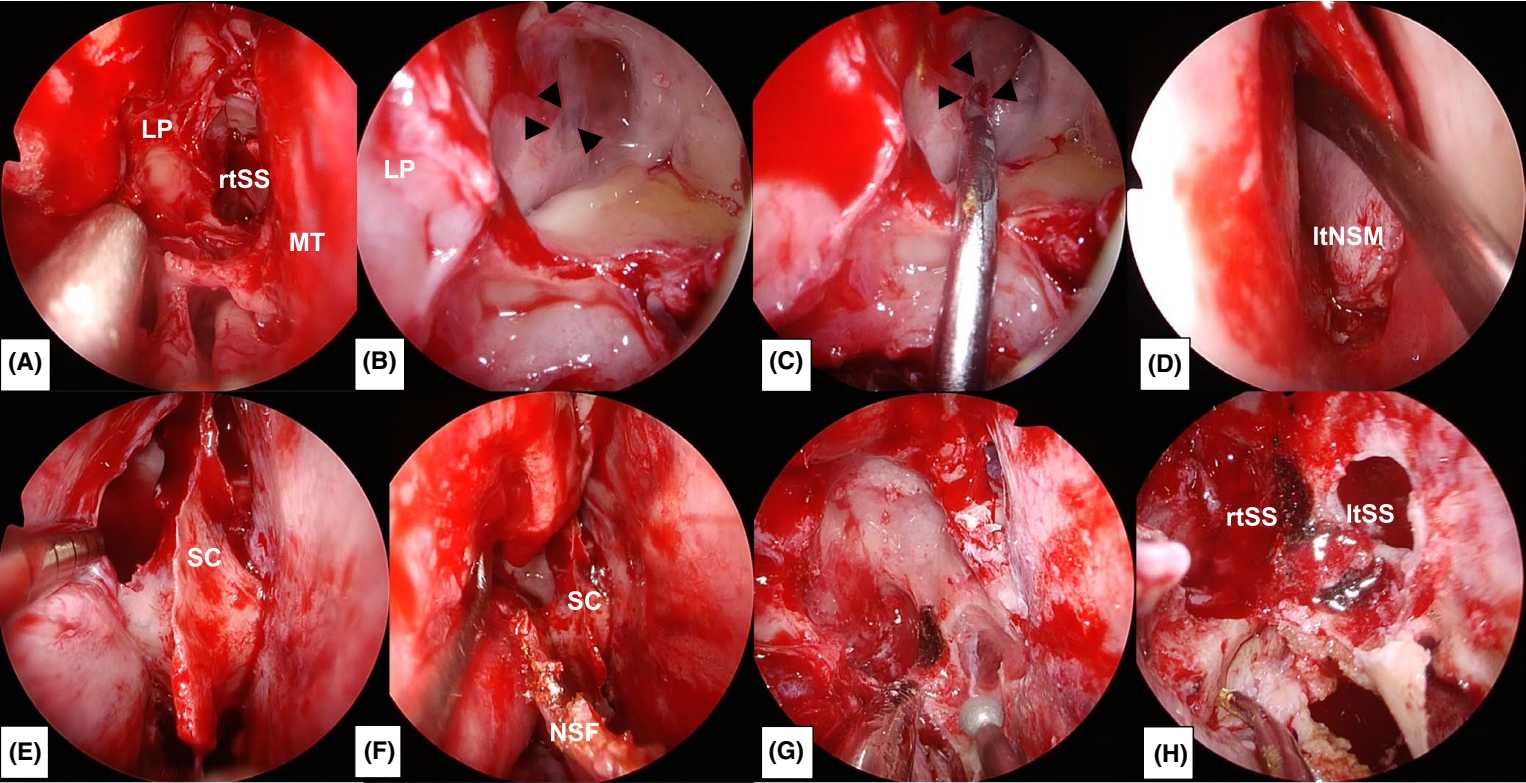
Intraoperative endoscopic findings accompanying the surgical procedure. The black arrowheads point to the fistula. (A) Right endoscopic sinus surgery is performed. (B) The fistula covered with the mucosa is identified at the lateral wall of the right sphenoid sinus with a 70‐degree telescope. (C) The fistula is further revealed after removal of a part of the mucosa and the CSF leak is confirmed with a 70‐degree telescope. (D) The left nasal septum mucosa is incised and elevated for the transseptal approach. (E) The sphenoid crest is identified at the posterior end of the nasal septum. (F) A rescue incision is made to the natural ostium of the right sphenoid sinus, parallel to the nasal floor. (G) The sphenoid crest is removed to expand the surgical field. (H) The surgical field is expanded laterally by removing the anterior wall of the sphenoid sinus. Abbreviations: LP, lamina papyracea; ltNSM, left nasal septum mucosa; ltSS, left sphenoid sinus; MT, middle turbinate; rtSS, right sphenoid sinus; SC, sphenoid crest

**FIGURE 5:**
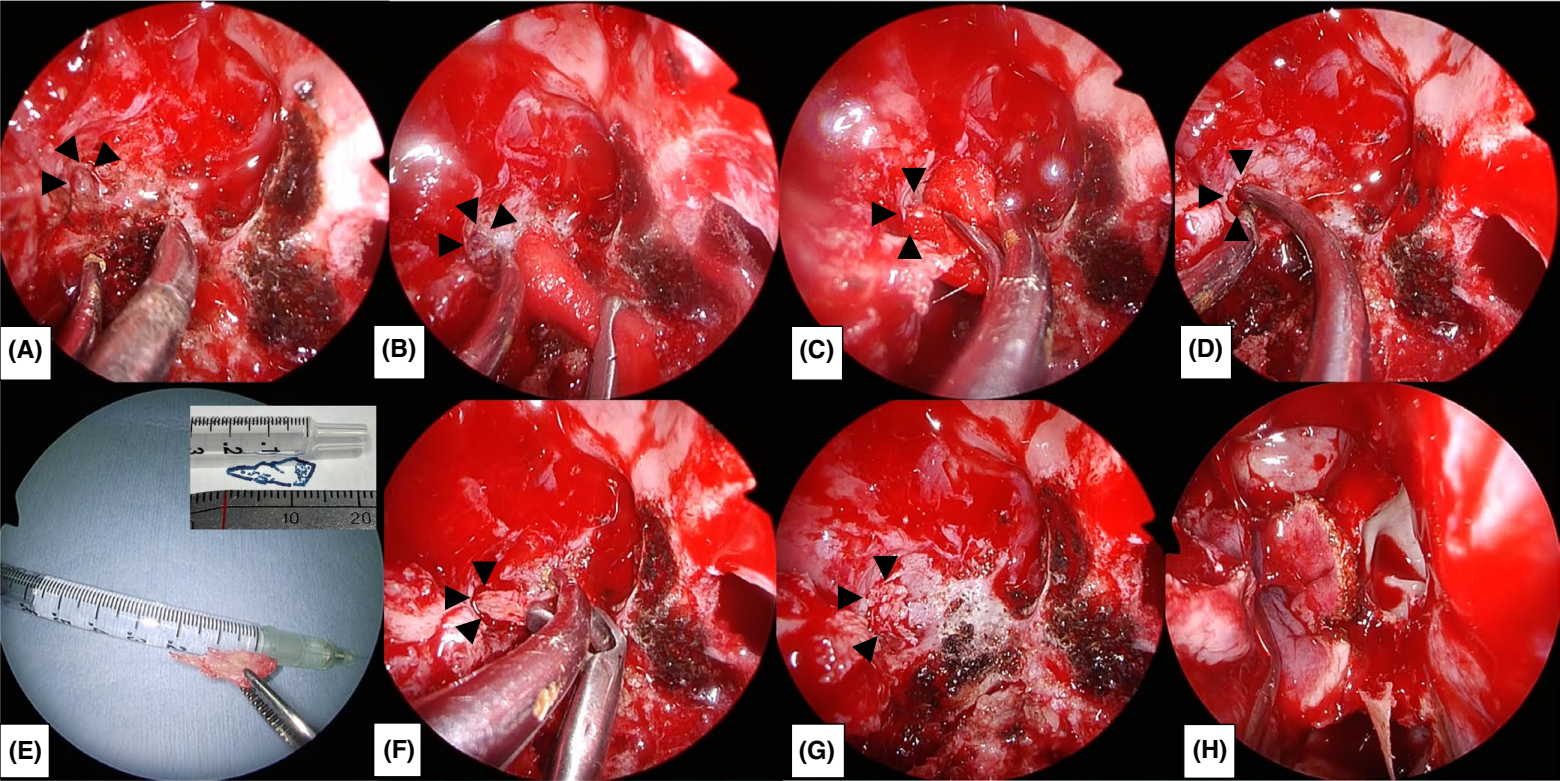
Intraoperative endoscopic findings accompanying the surgical procedure. The black arrowheads indicate the fistulas. E and H are findings with the 0‐degree telescope, and the remaining are findings with the 30‐degree telescope. (A) The arachnoid mater is identified in the fistula. (B, C, D) A collagen matrix is inserted into the fistula. (E) The bone is formed into a pile according to the size of the fistula. (F, G) The bone is inserted into the fistula in a pile‐driving manner. (H) The lateral wall of the sphenoid sinus is covered with a nasoseptal mucosal flap. Abbreviation: ltSS, left sphenoid sinus; NSF, nasoseptal mucosal flap

An otolaryngologist and a neurosurgeon performed the reconstruction surgery. First, right endoscopic sinus surgery was performed to open the sphenoid sinus via the ethmoid sinus (Figure 4A). The base of the right sphenoid sinus was observed with a 70‐degree telescope, and a fistula was identified on the lateral wall of the right sphenoid sinus (Figure 4B,C). Subsequently, bilateral sphenoid sinuses were opened using a transseptal approach (Figure 4D,E). To secure a wide surgical field and sufficient field of view, a rescue incision was made to the natural ostium of the right sphenoid sinus, parallel to the nasal floor, at the area where the olfactory epithelium did not exist (Figure 4F). The sphenoid crest was removed, and the surgical field was developed laterally so that the floor of the right sphenoid sinus could be fully manipulated (Figure 4G,H). The blood vessels and nerves in the right palatovaginal and Vidian canals were cauterized, and the surgical field was further expanded laterally to enable manipulation of the lateral wall of the right sphenoid sinus. The arachnoid mater was identified in the fistula after removal of the right sphenoid sinus mucosa (Figure 5A). A collagen matrix (DuraGen^TM^ Dural Graft Matrix [Integra Life Sciences, Plainsboro, NJ, USA]) was inserted into the fistula and laid against the dura mater (Figure 5B,C,D). The vomer bone was shaped into a pile and fitted into the fistula for rigid reconstruction of the skull base (Figure 5E,F,G). A multilayer reconstruction was performed by covering the outermost layer with a pedicle nasoseptal mucosal flap (Figure 5H).

At 6‐month follow‐up, no recurrence of CSF leakage or resorption of the inserted bone pile was observed (Figures 2B1‐3 and 3B). There were no complications, such as olfactory disturbances or nasal septal perforation, due to surgical invasion. There were no findings of intracranial infection or sinusitis.

## 3 DISCUSSION

We propose two clinical issues to be addressed in this case. First, rigid reconstruction was possible even for a narrow fistulous skull base defect. Second, thin‐slice HRCT images were effective in identifying the fistula.

The main feature of this procedure is the use of bone for rigid reconstruction. In this case, a multilayer reconstruction was performed, which included a collagen matrix laid against the dura mater as the first layer, bone pile fitted into the bone as the second layer, and a pedicle nasoseptal flap covering the bone as the third layer. Previously, for small defects, only soft reconstruction was performed with fascia or fat and rigid reconstruction was not recommended.^7^, ^8^, ^9^ However, since spontaneous CSF leakage is related to chronic intracranial hypertension,^2^, ^3^, ^4^, ^5^, ^6^ a strong reconstruction is preferable. In this case, the fistula was in the form of a narrow canal; therefore, we processed the vomer bone into a pile and inserted it in a pile‐driving manner. This technique provides a rigid bone‐based reconstruction for canalicular fistulas.

The fistula in this case was thin and canal‐like; thus, a 0.5‐mm slice HRCT was useful in identifying it. According to the previous reports, spontaneous CSF leakage in the sphenoid sinus has often been associated with meningoencephaloceles.^3^, ^10^, ^11^, ^12^ CT and MRI are commonly used imaging modalities, with a sensitivity of approximately 90%.^9^ However, in this case, CT and MRI of the brain did not raise suspicion of spontaneous CSF leakage. Even in the absence of the lateral recess of the sphenoid sinus and meningoencephalocele, it is important to recognize the possibility of CSF leakage via a canal‐like fistula and an ascending bacterial infection.

We performed reconstructive surgery of the fistula on the lateral wall of the sphenoid sinus using a transseptal approach. The transpterygoid approach is useful for observation and surgical manipulation of the defect in patients with a well‐developed lateral recess of the sphenoid sinus.^13^ Since the lateral recess of the sphenoid sinus was not observed in the current case, the surgical field was expanded laterally via the transseptal approach. We were able to observe and manipulate the defect area sufficiently using a 30‐degree telescope.

No complications were observed 6 months after surgery. However, it is necessary to investigate the possibility of resorption of the injected bone and recurrence of CSF leakage.

## 4 CONCLUSION

We demonstrated that rigid reconstruction with bone was possible for a narrow canal‐like fistula at the skull base. Thin‐slice HRCT was effective in identifying the fistula.

## CONFLICTS OF INTERESTS

There is no conflict of interest to disclose.

## AUTHOR CONTRIBUTION

Kosuke Takabayashi (K.T.) drafted the manuscript and substantially contributed to the conception and design of the research and interpretation of the data. Shuho Gotoh (S.G.) made a substantial contribution to the study conception and interpretation of data. Maeho Yamasaki (M.Y.) made a substantial contribution to the study conception. Katsumi Takizawa (K.T.) supervised the project, was responsible for data collection, and made a substantial contribution to the conception and design of the research and interpretation of the data. All authors made substantial contributions to the conception and design of the case report and interpretation of the data; revised the manuscript, approved it to be published, and agreed to be accountable for all aspects of the work in ensuring that questions related to the accuracy or integrity of any part of the work are appropriately investigated and resolved. Each author participated in this work for an appropriate portion of the content.

## ETHICAL APPROVAL

Approval for this case report was obtained from the Ethics Committee of the Japanese Red Cross Asahikawa Hospital (approval number: 202145–2).

## CONSENT

Written informed consent was obtained from the patient.

## Data Availability

The data that support the findings of this study are available from the corresponding author upon reasonable request.

## References

[1] Thakur JD , Manzi B , Savardekar AR , Singh MP , Menger R , Nanda A . Commentary: Maximilian Sternberg (1863–1934): the man behind Sternberg’s canal and his contribution to the modern‐day skull base anatomy and neuroscience‐historical vignette. Neurosurgery. 2018;83:E120‐E124. 29893958 10.1093/neuros/nyy242

[2] Illing E , Schlosser RJ , Palmer JN , Curé J , Fox N , Woodworth BA . Spontaneous sphenoid lateral recess cerebrospinal fluid leaks arise from intracranial hypertension, not Sternberg’s canal. Int Forum Allergy Rhinol. 2014;4(3):246‐250. 24407877 10.1002/alr.21262

[3] Hanz SZ , Lt A , Schmidt F , et al. Low incidence of true Sternberg’s canal defects among lateral sphenoid sinus encephaloceles. Acta Neurochir (Wien). 2020;162:2413‐2420. 32372133 10.1007/s00701-020-04329-2

[4] Settecase F , Harnsberger HR , Michel MA , Chapman P , Glastonbury CM . Spontaneous lateral sphenoid cephaloceles: anatomic factors contributing to pathogenesis and proposed classification. AJNR Am J Neuroradiol. 2014;35:784‐789. 24091443 10.3174/ajnr.A3744 PMC7965830

[5] Barañano CF , Curé J , Palmer JN , Woodworth BA . Sternberg’s canal: fact or fiction? Am J Rhinol Allergy. 2009;23(2):167‐171. 19401043 10.2500/ajra.2009.23.3290

[6] Koch CG , Grayson JW , Letter WBA . Letter: commentary: Maximilian Sternberg (1863–1934): the man behind Sternberg’s canal and his contribution to the modern‐day skull base anatomy and neuroscience‐historical vignette. Neurosurgery. 2021;88(5):E459‐E460. 33555008 10.1093/neuros/nyab014

[7] Konuthula N , Khan MN , Del Signore A , Govindaraj S , Shrivastava R , Iloreta AM . A systematic review of secondary cerebrospinal fluid leaks. Am J Rhinol Allergy. 2017;31:48‐56. 10.2500/ajra.2017.31.4487 29122076

[8] Sharma SD , Kumar G , Bal J , Eweiss A . Endoscopic repair of cerebrospinal fluid rhinorrhoea. Eur Ann Otorhinolaryngol Head Neck Dis. 2016;133:187‐190. 26776882 10.1016/j.anorl.2015.05.010

[9] Psaltis AJ , Schlosser RJ , Banks CA , Yawn J , Soler ZM . A systematic review of the endoscopic repair of cerebrospinal fluid leaks. Otolaryngol Head Neck Surg. 2012;147:196‐203. 22706995 10.1177/0194599812451090

[10] Samadian M , Moghaddasi H , Vazirnezami M , et al. Transcranial approach for spontaneous CSF rhinorrhea due to Sternberg’s canal intrasphenoidal meningoencephalocele: case report and review of the literature. Turk Neurosurg. 2012;22:242‐245. 22437302 10.5137/1019-5149.JTN.2902-10.1

[11] Bendersky DC , Landriel FA , Ajler PM , Hem SM , Carrizo AG . Sternberg’s canal as a cause of encephalocele within the lateral recess of the sphenoid sinus: a report of two cases. Surg Neurol Int. 2011;2:171. 22145089 10.4103/2152-7806.90034 PMC3229810

[12] Castelnuovo P , Dallan I , Pistochini A , Battaglia P , Locatelli D , Bignami M . Endonasal endoscopic repair of Sternberg’s canal cerebrospinal fluid leaks. Laryngoscope. 2007;117(2):345‐349. 17277632 10.1097/01.mlg.0000251452.90657.3a

[13] Yadav YR , Parihar V , Janakiram N , Pande S , Bajaj J , Namdev H . Endoscopic management of cerebrospinal fluid rhinorrhea. Asian J Neurosurg. 2016;11:183‐193. 27366243 10.4103/1793-5482.145101 PMC4849285

